# Is it possible to perform reverse sural artery flap on lower limbs where the main trunk of the peroneal artery is interrupted?

**DOI:** 10.1016/j.jpra.2025.01.012

**Published:** 2025-01-25

**Authors:** Go Nishizawa, Yuta Izawa, Kentaro Futamura, Masahiro Nishida, Yoshihiko Tsuchida, Naoya Inagaki, Mitsuru Saito

**Affiliations:** 1 Department of Trauma Centre, Shonan Kamakura General Hospital, 1370-1 Okamoto, Kamakura, Kanagawa, Japan; 2 Department of Orthopedic Surgery, The Jikei University School of Medicine, Tokyo, Japan

**Keywords:** Severe extremity trauma, Peroneal artery, Reverse sural artery flap, Flap necrosis, Infection, Delay procedure

## Abstract

As blood flow to the reverse sural artery flap (RSAF) occurs via the peroneal artery, the health of the peroneal artery is generally considered important. In severe limb injuries, the main trunk of the peroneal artery is often disrupted at the fracture site, which brings uncertainty in the use of RSAF. We hypothesized that RSAF could be used even in cases where the main trunk of the peroneal artery is interrupted if retrograde blood flow from the plantar side forms a communication network with the superficial sural artery. Therefore, we performed RSAF in cases where the blood flow of the communicating branch could be confirmed. This study included patients who underwent RSAF when the main trunk of the peroneal artery was interrupted by trauma. Patient demographics, characteristics of injury, and treatment course were obtained from medical records. The outcome of this study included flap survival and complications, such as partial necrosis or flap infection. Five limbs that underwent RSAF met the inclusion criteria. In all cases, the skin flaps survived and soft tissue reconstruction was completed. Infection and partial necrosis of the skin flaps were observed in 2 cases each. This study showed that the RSAF can be used, even in cases where the main trunk of the peroneal artery had been interrupted, if retrograde blood flow from the plantar side forms a communication network with the superficial sural artery.

## Introduction

Reverse sural artery flap (RSAF) is commonly used to reconstruct soft tissue defects in the distal leg and foot.^1^^,^^2^ This flap does not require the use of microsurgical techniques, blood flow is relatively stable, and main artery of the lower leg can be preserved.^3^, ^4^, ^5^, ^6^, ^7^, ^8^, ^9^

The superficial sural artery runs alongside the sural nerve and lesser saphenous vein and gives off minute branches into the skin in the distal two-thirds of the lower leg.^10^ The peroneal artery branches off from the popliteal artery, and while giving off multiple perforators, descends under the skin. In the distal part of the lower leg, the superficial sural artery and perforators of the peroneal artery anastomose to form a communicating branch.^11^ The RSAF is a skin flap nourished by retrograde blood flow to the superficial sural artery via this communicating branch. Therefore, maintaining the integrity of the communicating branches and superficial sural arteries is essential.

As the blood flow to the RSAF occurs via the peroneal artery, the health of the peroneal artery is generally considered important. RSAF is can be carried out in the lower limbs where the anterior and posterior tibial arteries are absent or interrupted, and only the peroneal artery remains; this highlights the importance of the peroneal artery in RSAF.^12^ However, in severe limb injuries involving lower leg fractures and soft tissue defects, the main trunk of the peroneal artery is often interrupted at the fracture site, and this causes uncertainty in using RSAF.

We hypothesized that RSAF could be used even in cases where the main trunk of the peroneal artery had been interrupted, if retrograde blood flow from the plantar side forms a communication network with the superficial sural artery. Thus, we attempted soft tissue reconstruction using RSAF in cases where the blood flow of the communicating branch could be confirmed.

This study aimed to report the treatment outcomes of using RSAF in cases where the main trunk of the peroneal artery was severed due to trauma.

## Materials and Methods

This study included patients who underwent RSAF when the main trunk of the peroneal artery was damaged by trauma between September 2022 and September 2024. Approval for this study was obtained from all patients.

Patient demographics were obtained from the medical records. The fracture type, health of the trifurcation of the lower extremity, location of soft tissue defects, and duration between elevation of the skin flap and migration were investigated. The primary outcome was flap survival. Secondary outcomes, including the presence or absence of complications, such as partial necrosis or flap infection, were investigated. Associated fractures were evaluated using radiography and computed tomography (CT). The need for soft tissue reconstruction and method of soft tissue reconstruction were determined by each surgeon. In cases where soft tissue reconstruction was considered necessary, CT angiography of the lower leg was performed to evaluate the health of the trifurcation of the lower extremity. The criteria for determining whether RSAF could be performed were as follows: the peroneal artery was partially disrupted at the fracture site, but the distal portion was contrasted by retrograde blood flow from the plantar side; a healthy lesser saphenous vein could be visualized using ultrasound; and the communicating branches of the peroneal and superficial sural arteries could be detected using Doppler. Considering the possibility of unstable circulation compared with a healthy peroneal artery trunk, RSAF was performed in all patients using the stepwise delay method.^13^^,^^14^ Complications were determined by the primary surgeon, and surgical intervention was performed as needed. In cases of partial necrosis, debridement of the necrotic area of the flap was performed and flap placement was corrected. In the case of infection, the patient was treated with frequent irrigation in the operating room, systemic administration of antibiotics, and high-concentration local antibiotic therapy.^14^

## Results

In this study, 5 limbs met inclusion criteria. The average age was 58.6 ± 17.4 years, and all were men. The mechanism of injury was traffic accident in 2 cases, fall in 2 cases, and crush injury in 1 case. The fracture type was tibiofibular diaphyseal fracture in 3 cases and ankle fracture in 2 cases. In 3 cases, the site of the soft tissue defect was the distal lateral side of the lower leg; in 1 case, it was the distal medial side of the lower leg; and in 1 case, it was the heel. The anterior and posterior tibial arteries were intact in 4 patients, and only the anterior tibial artery remained in 1 patient. The average time from skin flap elevation to transfer was 7.4 ± 3.3 days. In all cases, the skin flaps survived and soft tissue reconstruction was completed. Infection was observed in 2 cases, and partial necrosis of the skin flaps was observed in 2 cases (Table 1).

**Table 1:** Patient demographics, characteristics of injury, and treatment outcomes.

Case	Age, y	Sex	Mechanism of injury	Fracture type	Health of trifurcation			Location of soft tissue defect	Duration between flap elevation and migration, days	Flap survival	Complications
					PA	ATA	PTA				
1	71	M	Fall injury	Tibiofibular diaphyseal	×	°	°	Distal lateral lower leg	9	°	None
2	27	M	Traffic accident	Ankle	×	°	°	Distal lateral lower leg	2	°	None
3	56	M	Crush injury	Tibiofibular diaphyseal	×	°	×	Heel	7	°	Partial necrosis
4	62	M	Traffic accident	Tibiofibular diaphyseal	×	°	°	Distal medial lower leg	7	°	Infection
5	77	M	Fall injury	Ankle	×	°	°	Distal lateral lower leg	12	°	Partial necrosis, infection

M: Male

PA: Peroneal artery

ATA: Anterior tibial artery

PTA: Posterior tibial artery

×: Disruption of major artery

°: Survival of major artery or flap

### Case presentation

#### Case 1

**Figure 1:**
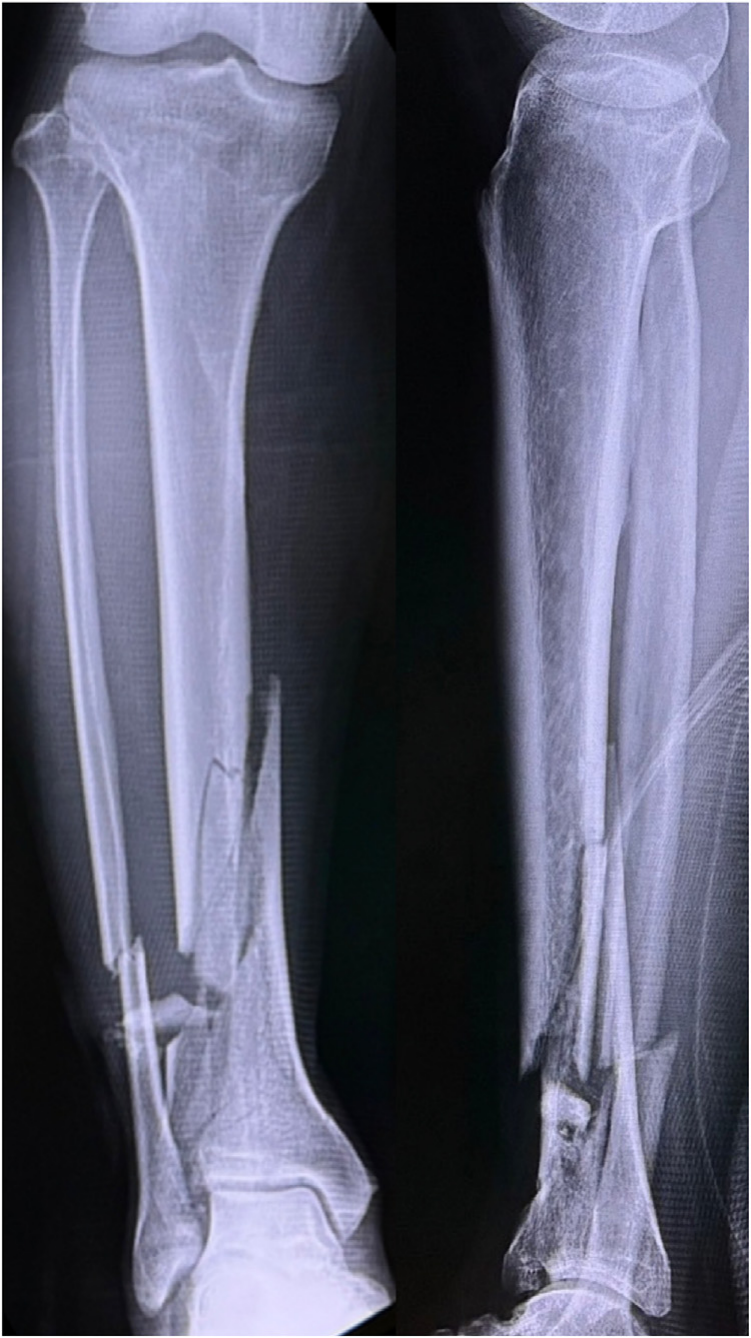
Plain radiograph at the time of injury.

**Figure 2:**
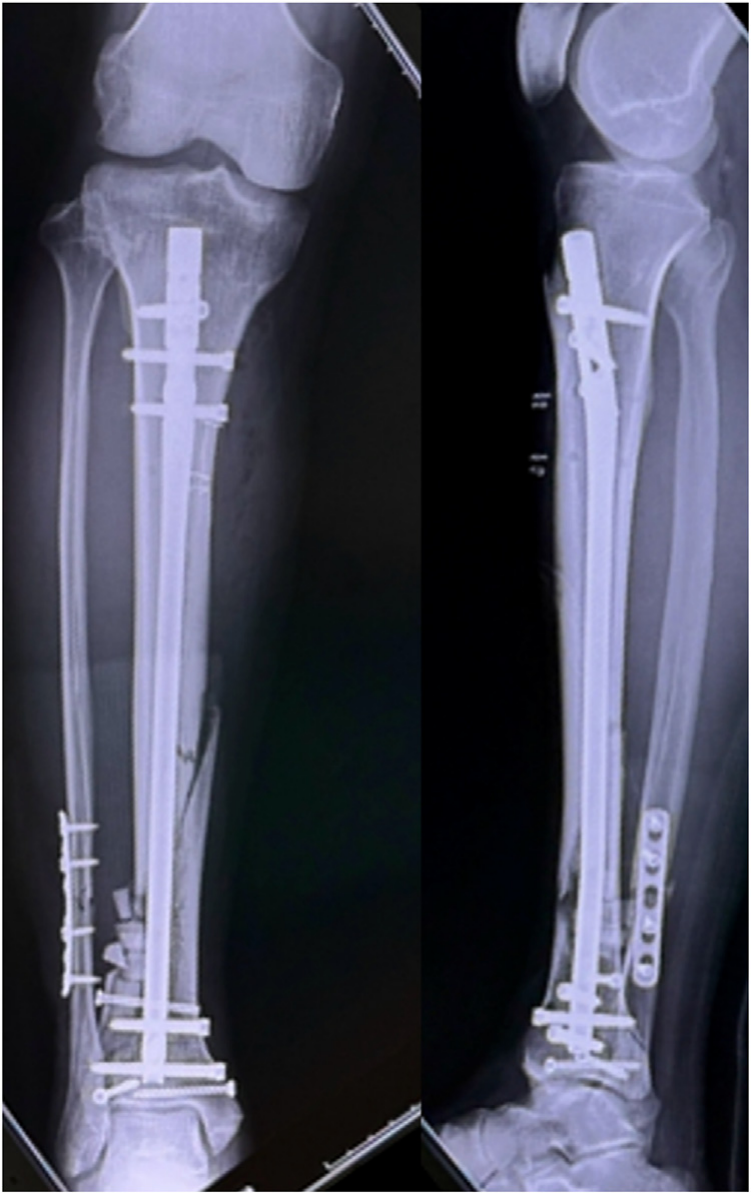
Plain radiograph after osteosynthesis using intramedullary nail and plate.

**Figure 3:**
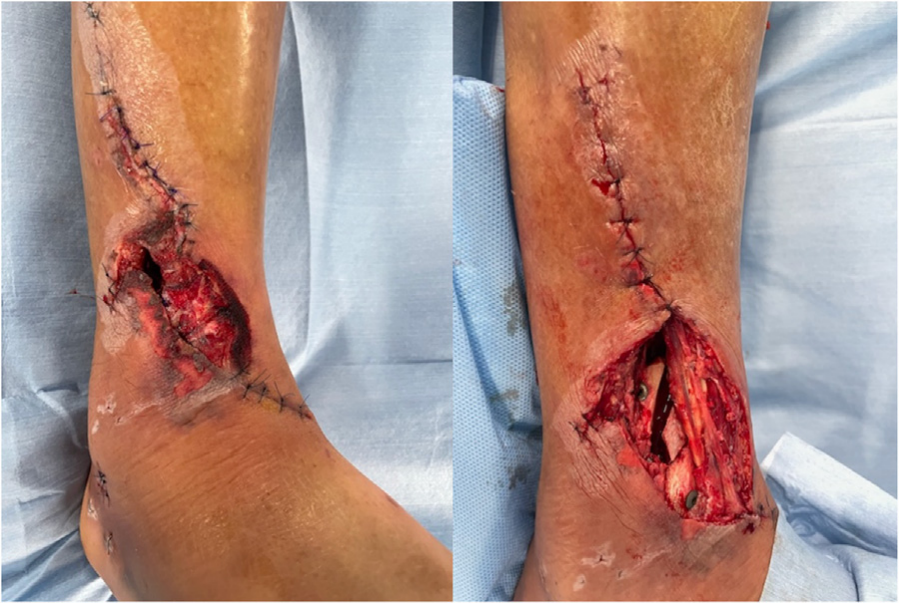
Skin around the original open wound is necrotic. After debridement of necrotic soft tissue, the tibia and implants are exposed.

**Figure 4:**
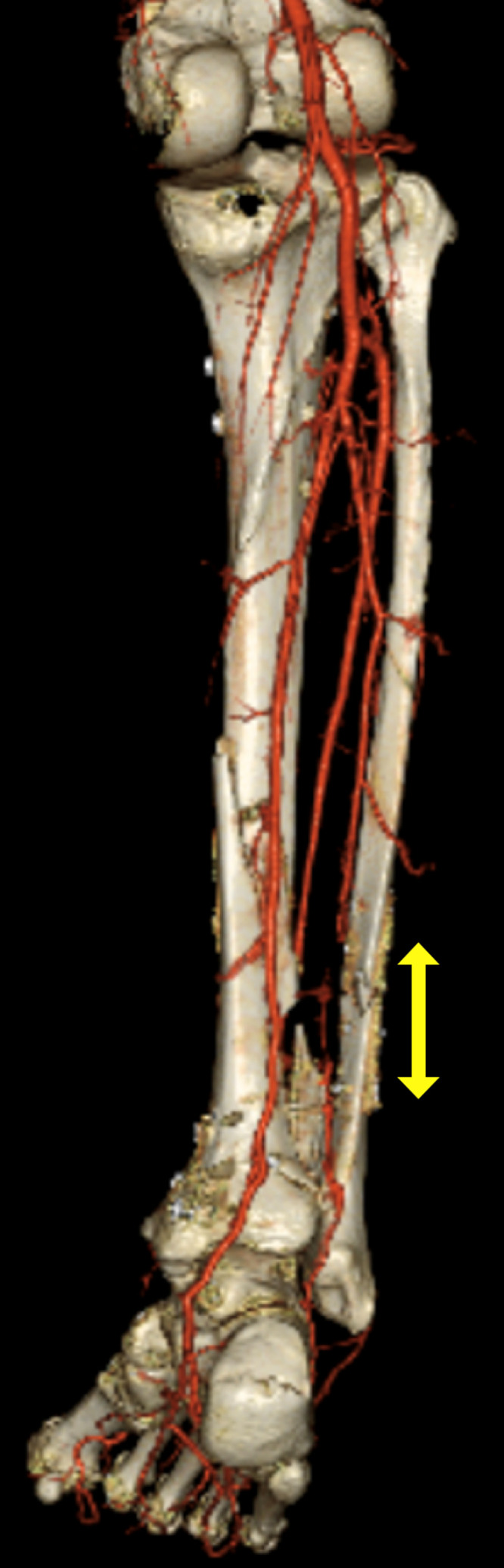
Contrast-enhanced CT at the time of transfer to our hospital. Although the anterior and posterior tibial arteries were intact, the peroneal artery was interrupted at the fracture site (yellow arrow).

**Figure 5:**
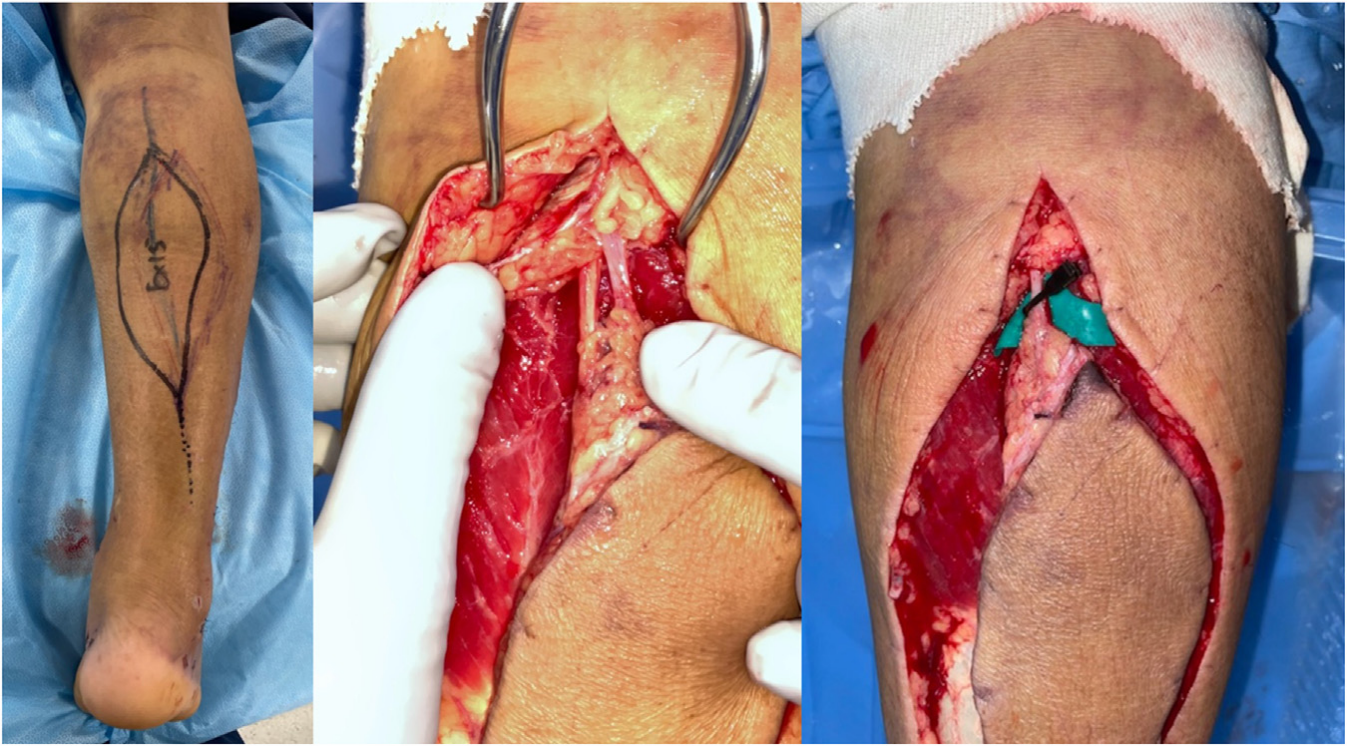
Reverse sural artery flap (RSAF) was elevated and stepwise delay procedure was performed.

**Figure 6:**
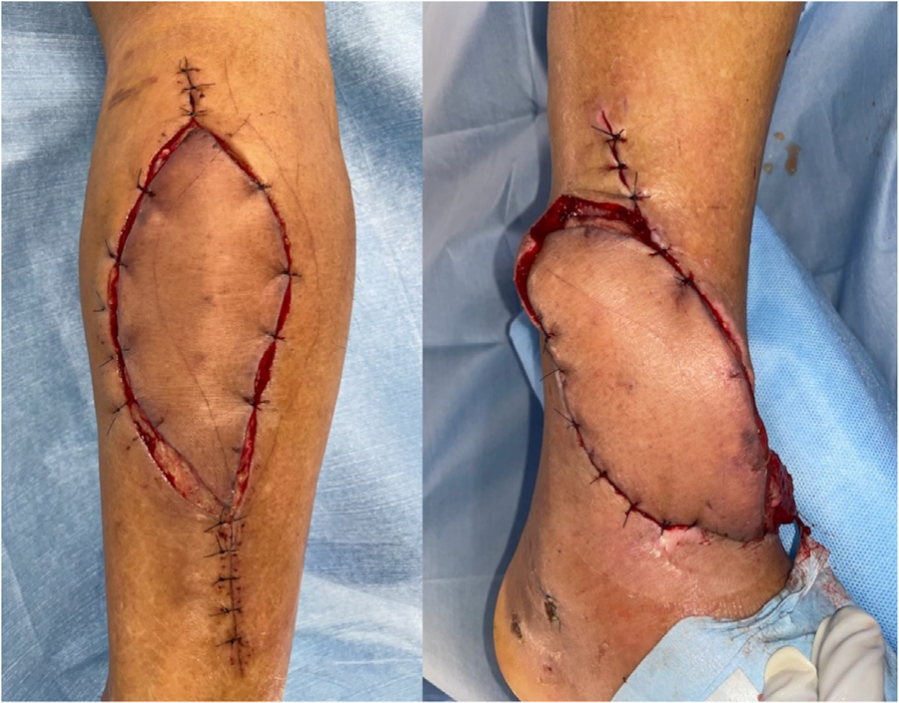
Skin flap was moved to soft tissue defect area 9 days after elevation.

**Figure 7:**
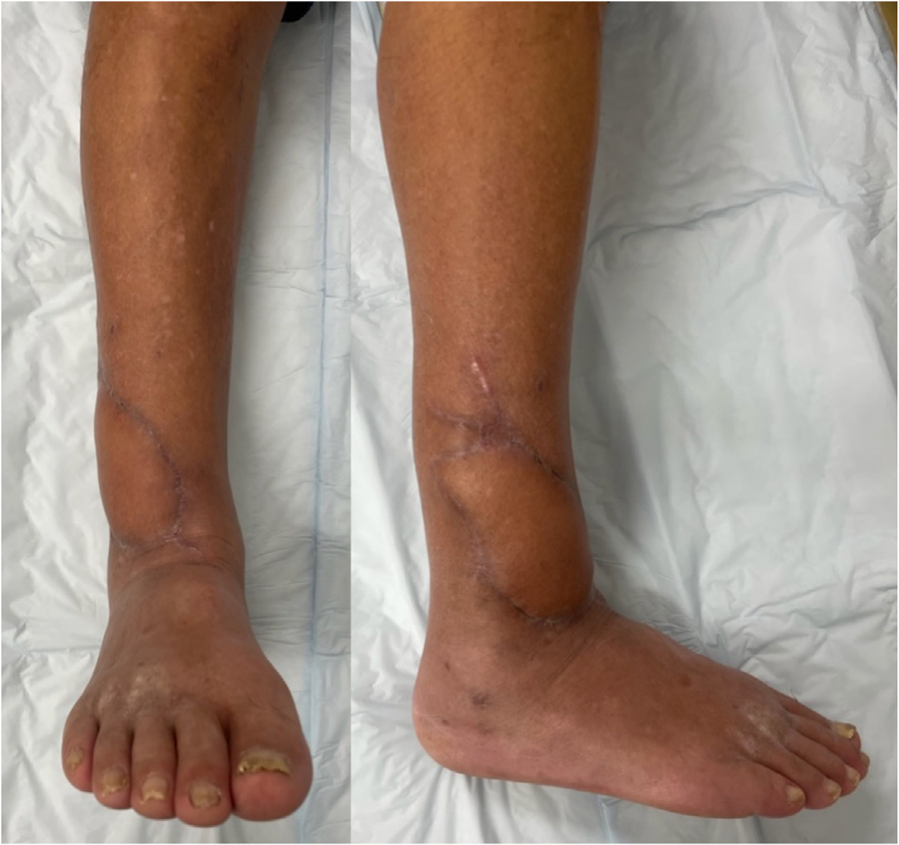
Skin flap survived without any complications.

A 71-year-old man presented with diabetes. The patient had fallen from a ladder and was injured. Subsequently, the patient was transported to another hospital. An open wound was observed on the anterolateral side of the right lower leg. The patient was diagnosed with a Gustilo-Anderson classification type 3A open fracture of the distal tibia and diaphysis of the fibula (Figure 1). On the day of the injury, external fixation was performed, and the open wound was cleaned and closed. On the 11th day after the injury, internal fixation was performed using an intramedullary nail for the distal tibia fracture and a plate for the fibula diaphyseal fracture (Figure 2). Fifteen days after the injury, the skin around the original open wound became necrotic. Debridement was performed, which resulted in a defect in the soft tissue, and the patient was transferred to our hospital (Figure 3). On contrast-enhanced CT, the anterior and posterior tibial arteries were intact; however, the peroneal artery was interrupted at the fracture site (Figure 4). On the 17^th^ day after the injury, RSFA was elevated and step wise delay procedure was performed (Figure 5). On the 9^th^ day after elevation, the skin flap was moved to the soft tissue defect area (Figure 6). The skin flap survived without any complications (Figure 7).

#### Case 2

A 27-year-old man with no history of illness was injured when his truck collided with another vehicle and sustained injury to his left lower leg. An open wound was observed on the distal anterior lateral side of the left lower leg. Contrast-enhanced CT showed that the anterior and posterior tibial arteries were intact, but the peroneal artery was interrupted at the fracture site. The patient was diagnosed with a Gustilo-Anderson classification type 3 B ankle joint fracture and underwent intramedullary pinning for the fibula and wound closure. On the 17th day after the injury, debridement was performed on the necrotic wound edges, resulting in a soft tissue defect. On the 20th day after injury, the RSAF was elevated. On the 2nd day after elevation, the skin flap was moved to the soft tissue defect area. The skin flap survived without any complications.

#### Case 3

A 56-year-old man with no relevant medical history was injured when he dropped a heavy object onto his right lower leg. The patient was diagnosed with right tibial diaphysis fracture. Contrast-enhanced CT showed that the anterior tibial artery was intact, but the posterior tibial and peroneal arteries were interrupted at the fracture site. Two days after the injury, an intramedullary nail was used to perform osteosynthesis of the tibial diaphyseal fracture, and intramedullary pinning was performed for the fibula. On the 12th day after the injury, a pressure ulcer was observed on the right heel. On the 14th day after injury, partial necrosis was observed in the pressure sore area, and debridement was performed, resulting in a soft tissue defect. On the 25th day after the injury, the RSAF was elevated, and the skin flap was moved 7 days after elevation. Partial necrosis of the flap owing to congestion was observed after the skin flap was moved. Debridement of the necrotic flap area was performed, flap placement was corrected, and the skin flap eventually survived.

#### Case 4

A 62-year-old man with no relevant medical history was injured after falling off a motorcycle. He was diagnosed with a Gustilo-Anderson classification type 3B open fracture of the tibia and fibula, with an open wound on the medial side of the lower leg. Contrast-enhanced CT revealed that the anterior and posterior tibial arteries were intact; however, the peroneal artery was interrupted at the fracture site. External fixation was performed on the day of the injury, and 3 days after the injury, osteosynthesis was performed for the tibial diaphyseal fracture. Although the wound was closed, necrosis of the wound edges resulted in soft tissue defects. On the 25th day after the injury, the RSAF was elevated and skin flap was moved 7 days after elevation. Infection occurred after the skin flap was moved; however, it improved with frequent irrigation, systemic administration of antibiotics, and high-concentration local antibiotic treatment, and the skin flap eventually survived.

#### Case 5

A 77-year-old man presented with diabetes and arteriosclerosis. He had fallen and injured himself while walking and was transported to another hospital. The patient was diagnosed with an ankle fracture. On day 2 after the injury, osteosynthesis with plates and screws was performed; however, 90 days after the surgery, redness was observed in the lateral plate area. The plate was removed, skin was debrided, and patient was transferred to our hospital with a soft tissue defect in the lateral part of the ankle joint. On contrast-enhanced CT, the anterior and posterior tibial arteries were intact; however, the peroneal artery was interrupted at the fracture site. Four days after the transfer, the implants in the medial and posterior malleolus were removed, and 14 days after transfer, the RSAF was elevated. Twelve days after elevation, the skin flap was removed. After the skin flap was moved, partial necrosis of the flap surface due to congestion was observed. In addition, the infection improved with frequent irrigation, systemic administration of antibiotics, and high-concentration local antibiotic treatment, and the skin flap eventually survived. Full-thickness skin grafting was performed in the area of superficial necrosis of the flap.

## Discussion

This is the first study to examine the feasibility of RSAF in cases where the main trunk of the peroneal artery is interrupted. As the skin flaps survived in all 5 cases, RSAF may be used even if the main trunk of the peroneal artery is interrupted, as long as the communicating branch is healthy. Partial necrosis of the skin flaps occurred in 2 cases, which was assumed to be triggered by congestion of the skin flaps after they had been moved. Although there was a risk of ischemia due to the interruption of antegrade blood flow into the communicating branch from the proximal side of the peroneal artery, it is possible to sufficiently nourish the skin flap with blood flow from the plantar side via the communicating branch. We used the stepwise delay method in all cases, considering the possibility that the blood supply may be unstable compared to when the main trunk of the peroneal artery is still present. In this technique, the proximal vascular pedicle is left in place when the skin flap is elevated and the arteries and veins in the vascular pedicle are blocked using vascular clips. If the skin flap appeared to be ischemic or congested due to blockage of the proximal vascular pedicle, the clips were removed, and the blockage was re-established using vascular clips 2–3 days later. When the skin flap no longer showed signs of ischemia or congestion, the blood flow in the skin flap became independent, and the proximal vascular pedicle could be resected. Using this technique, the timing at which it is safe to move the skin flap can be judged while simultaneously strengthening the blood flow via the communicating branches and venous network. It is unclear whether the skin flap can be safely moved without using the delay method in cases where the main trunk of the peroneal artery has been interrupted. In both cases, the period from injury to skin flap elevation was long, and the cause of infection was considered to be the wound that remained open for a long duration. Interruption of the main trunk of the peroneal artery is not considered to be related to the onset of infection.

If RSAF can be carried out in cases where the main trunk of the peroneal artery has been disrupted, the technically difficult free flap surgery can be avoided, making open fracture treatment of the lower leg simpler and easier. In addition, in preoperative planning for soft tissue reconstruction, it is necessary to use contrast-enhanced CT to evaluate the vascular injury location and recipient vessel condition for free flaps. In patients who cannot undergo contrast-enhanced imaging because of allergies or renal dysfunction, it is not possible to assess the vascular injury location or recipient vessel condition. However, if it is possible to visualize the lesser saphenous vein using ultrasound and detect the communicating branch using Doppler, RSAF can be an extremely important treatment option.

This study has some limitations. First, our study was retrospective, and because the targeted trauma was diverse, there were no firm criteria for selecting the surgical methods. In addition, the number of cases was small because we targeted a rare condition of severe limb trauma, in which the main trunk of the peroneal artery is interrupted. This study only suggests the possibility of using RSAF in such conditions; however, further research with a larger sample size is necessary to confirm its safety and efficacy.

## Conclusion

We reported the treatment outcomes of 5 cases, wherein RSAF was performed despite the interruption of the main trunk of the peroneal artery due to trauma. In all cases, the skin flap survived and RSAF was possible if the communicating branch between the peroneal and superficial sural arteries remained intact.

## Informed consent

Informed consent was obtained from the patient in this study.

## CRediT authorship contribution statement

**Go Nishizawa:** Conceptualization, Investigation, Writing – original draft. **Yuta Izawa:** Methodology, Writing – review & editing. **Kentaro Futamura:** Supervision. **Masahiro Nishida:** Data curation, Supervision. **Yoshihiko Tsuchida:** Project administration. **Naoya Inagaki:** Supervision. **Mitsuru Saito:** Project administration.

## Declaration of competing interest

None.
